# Synthesis and Antibacterial Activity of Ionic Liquids and Organic Salts Based on Penicillin G and Amoxicillin hydrolysate Derivatives against Resistant Bacteria

**DOI:** 10.3390/pharmaceutics12030221

**Published:** 2020-03-02

**Authors:** Ricardo Ferraz, Dário Silva, Ana Rita Dias, Vitorino Dias, Miguel M. Santos, Luís Pinheiro, Cristina Prudêncio, João Paulo Noronha, Željko Petrovski, Luís C. Branco

**Affiliations:** 1 Ciências Químicas e das Biomoléculas (CQB) e Centro de Investigação em Saúde e Ambiente (CISA), Escola Superior de Saúde do Instituto Politécnico do Porto, 4400-330 Porto, Portugal; anaritadias3@gmail.com (A.R.D.); solchemar@gmail.com (V.D.); cps@estsp.ipp.pt (C.P.); 2 LAQV-REQUIMTE, Departamento de Química e Bioquímica, Faculdade de Ciências, Universidade do Porto, Rua do Campo Alegre 687, 4169-007 Porto, Portugal; 3 LAQV-REQUIMTE, Departamento de Química, Faculdade de Ciências e Tecnologia da Universidade Nova de Lisboa, 2829-516 Caparica, Portugal; dmv.silva@campus.fct.unl.pt (D.S.); miguelmsantos@fct.unl.pt (M.M.S.); l.pinheiro@campus.fct.unl.pt (L.P.); jpnoronha@fct.unl.pt (J.P.N.); 4 i3S, Instituto de Inovação e Investigação em Saúde, Universidade do Porto, 4099-002 Porto, Portugal

**Keywords:** active pharmaceutical ingredients-ionic liquids and organic salts (API-OSILs), penicillin G and amoxicillin hydrolysate derivatives, sensitive bacteria, resistant bacteria

## Abstract

The preparation and characterization of ionic liquids and organic salts (OSILs) that contain anionic penicillin G [secoPen] and amoxicillin [*seco*-Amx] hydrolysate derivatives and their in vitro antibacterial activity against sensitive and resistant *Escherichia coli* and *Staphylococcus aureus* strains is reported. Eleven hydrolyzed β-lactam-OSILs were obtained after precipitation in moderate-to-high yields via the neutralization of the basic ammonia buffer of antibiotics with different cation hydroxide salts. The obtained minimum inhibitory concentration (MIC) data of the prepared compounds showed a relative decrease of the inhibitory concentrations (RDIC) in the order of 100 in the case of [C_2_OHMIM][*seco*-Pen] against sensitive *S. aureus* ATCC25923 and, most strikingly, higher than 1000 with [C_16_Pyr][*seco*-Amx] against methicillin-resistant *Staphylococcus aureus* (MRSA) ATCC 43300. These outstanding in vitro results showcase that a straightforward transformation of standard antibiotics into hydrolyzed organic salts can dramatically change the pharmaceutical activity of a drug, including giving rise to potent formulations of antibiotics against deadly bacteria strains.

## 1. Introduction

Bacterial resistance to antibiotics has been increasing in Europe over the last few years [1,2,3]. New classes of antibiotics have not been introduced recently [4,5,6,7], and, thus, more resistances to old drugs are developing daily [8,9,10]. Recent efforts and huge investments being made in this field by big pharma companies such as GlaxoSmithKline, Merck, Pfizer and Wyeth [3,4,5,11,12] have had disappointing returns from their R&D departments, including clinical trials. This is a significant factor to allocate anti-infective R&D resources into other fields of investigation and thus remain highly competitive [3,4,5,13]. Considering the disappointing results on genomics and the exodus of big pharma, the problem of bacteria resistance has continued to evolve, reaching alarming dimensions [3,8,10].

For the last 12 years, organic salts and ionic liquids (OSILs) from active pharmaceutical ingredients (APIs), or simply API-OSILs [14,15,16,17,18,19,20,21,22], have been studied at the academic level [14,18,19,23,24,25]. Ionic liquids (ILs) are salts with melting points below 100 °C (some of them are liquid at room temperature) that result from the pairing of organic cations with organic and inorganic anions [14,20,23]. When the melting point is above 100 °C, these compounds are simply designated by organic salts [24]. Nowadays, there is a significant increase in the scope of both the physical and chemical properties of OSILs [19,26,27,28,29], and, thus, their application in several topics of science and technology is currently being studied [15,16,17,18,20,23,26].

In the case of API-OSILs, it is known that the interaction between an ionic API with selected counter-ions may significantly improve the pharmaceutical activity of the former [17,30,31,32]. In addition, this combination may also boost the stability and solubility of the API in physiological media, as well as enhance the bioavailability and modify the pharmacokinetics and delivery mode of the drug [21,27,28,29,33,34,35]. Consequently, the biopharmaceutics drug classification system (BCS) for API-ILs can be significantly modified in comparison with the parent drugs [24], meaning that this new salt of the old API can be treated as a new chemical entity and thus be independently patented [17,26,30,36]. Furthermore, the polymorphism of a given API can be severely mitigated or even eliminated if it becomes liquid, hence tackling one of the most important problems in the pharmaceutical industry that can dramatically alter a drug’s solubility and dosages [26,30,37,38,39,40]. In fact, solid forms of drugs can suffer from several limitations such as low solubility, polymorphic conversion, and low bioavailability [20,26,36,41].

The inherent properties of ILs could be of extreme importance to overcome such difficulties of solid form drugs [20,36,41]. Recent works have shown that API-OSILs possess many attractive properties when compared to conventional drugs [14,16,20,23]. Our group recently studied the relevant pharmacological properties of ampicillin- and primaquine-based API-OSILs such as water solubility, the octanol–water partition coefficient, the hexadecylphosphocholine (HDPC) micelle–water partition coefficient, and critical micelle concentration [14,15,16,17,30]. In the case of ampicillin-based API-OSILs, the data are clearly consistent with a greater potential of API-ILs in comparison with the parent API, specifically regarding their solubility in water, as well as more specific properties such as membrane affinity and permeation. In fact, the accurate selection of the organic cation allows for the fine-tuning of some important physical and thermal properties like water solubility, membrane permeation, melting point, and thermal stability [17]. In another study, we found that primaquine API-OSILs had a particular affinity to intercalate negatively-charged lipid bilayers (membrane models of *Plasmodium* infected erythrocyte) and also, to a lesser extent, zwitterionic lipid bilayers (membrane models of healthy cells), in comparison with the parent drug [14,42].

A large quantity of recent communications and reviews have referred to the toxicity and activity of ILs against microorganisms and cell cultures, especially antimicrobial activity, and as novel forms of bioactive materials and as drug delivery systems [14,20,43,44,45,46]. Recently, OSILs have been studied to fight multi-drug resistance [16,47] and as agents for microbial biofilms [32,45,48,49,50,51], and they have shown a potent, broad spectrum activity against a variety of clinically significant microbial pathogens, including methicillin-resistant *Staphylococcus aureus* (MRSA) [32,49,52]. The 2011 outbreak of multi drug-resistant *Escherichia coli* O104 in Germany as well as as Gram-negative Enterobacteriaceae due to presence of the New Delhi metallo-β-lactamase [53,54] are examples of an increasingly documented major public health problem.

Therefore, there is an increasing demand to develop new drugs to address multi-resistant infections and to develop more efficient tools so that new resistances are not developed. In this context, the results up to this point given by API-OSILs have followed these demands [20].

Thus, following our work on ampicillin [16,17,18] and fluoroquinolone-based API-OSILs [30], we herein describe the synthesis of ILs based on amoxicillin and penicillin G through a synthetic strategy that was optimized by us in the past for the preparation of ampicillin-OSILs [18]. The synthetic method consists on the deprotonation of an API with different hydroxides of organic cations in an ammonium buffer (buffer-controlled reaction procedure) [18]. In all cases, the antibiotic API is combined as an anion with organic cations that contain imidazolium, ammonium, phosphonium and pyridinium structures.

## 2. Results and Discussion

### 2.1. Chemistry

**Figure 1:**
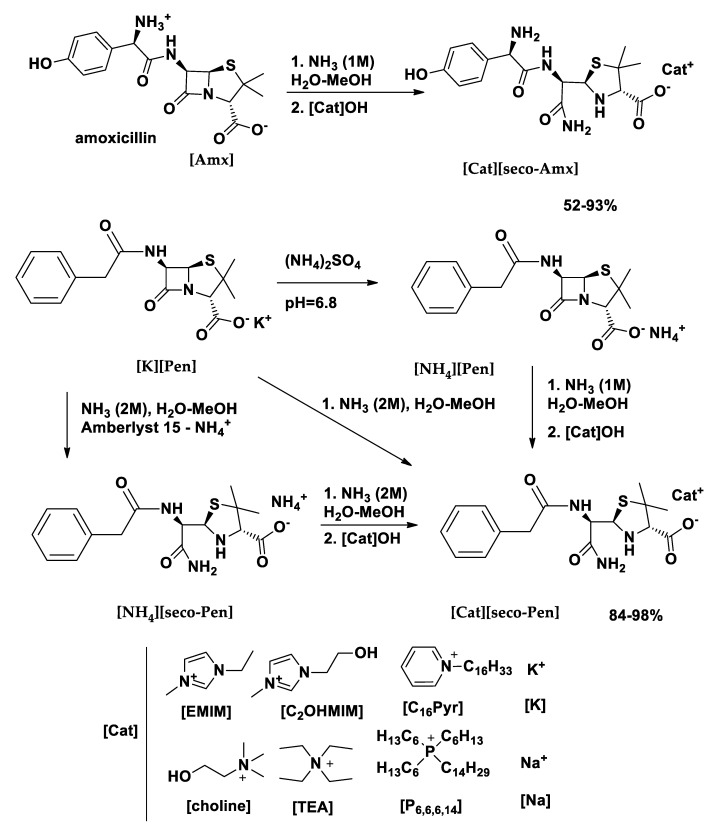
Schematic synthetic methodology for the preparation of metallic active pharmaceutical ingredients (API) salts and [*seco*-Amx] and [*seco*-Pen] ionic liquids and organic salts (OSILs).

Ionic liquids and organic salts based on the ammonia hydrolysate anion of penicillin G and amoxicillin (α-amide of benzyl penicilloic acid and of amoxicillin penicilloic acid, respectively), abbreviated here as [*seco*-Pen] and [*seco*-Amx], respectively, were prepared by an ammonia buffer reaction procedure that was recently developed by us for the synthesis of ampicillin API-OSILs [18]. Our original idea was to test a new method for preparation of API-OSILs based on parent penicillin and amoxicillin in the anionic form. However, due to ammonolysis (β-lactam ring opening with the formation of an amide group) of amoxicillin and penicillin G under the employed reaction conditions, ionic liquids and organic salts of [*seco*-Pen] and [*seco*-Amx] anions were prepared instead (see Figure 1). The prefix *seco* (Latin verb secare) is used in antibiotics nomenclature [55], and it means to cut. Two penicilloic acids of penicillin G and amoxicillin are already well known in the literature [56,57,58], and some of their stable amides—products of the amino- and ammonolysis of β-lactam ring of parent antibiotics—have also already been described [59,60,61].

While amoxicillin (Amx) was used as provided (in trihydrate form), penicillin G, which was supplied as a potassium salt ([K][Pen]), was previously converted to the corresponding ammonium salt [NH_4_][Pen] by following the procedure of Brown et al. [62]. The experimental procedure consisted on the reaction of hydroxide of the selected cations with amoxicillin and penicillin ammonium salt. Halide (chloride, bromide) salts of selected organic cations were converted into the corresponding hydroxides on Amberlite resin (OH form), and the highly basic solution that was obtained was then added to the solution of the API in an aqueous ammonia buffer in order to provide the compounds [18]. [Na][*seco*-Amx] was prepared through a reaction with sodium hydroxide (instead of organic hydroxide), while a [K][*seco*-Pen] derivative was directly obtained by ammonolysis from [K][Pen].

Hydrolyzed amoxicillin (*seco*-Amx) and penicillin G (*seco*-Pen) derivatives in the anionic form were combined with the following organic cations (see Figure 1): 1-ethyl-3-methylimidazolium [EMIM], 1-hydroxy-ethyl-3-methylimidazolium [C_2_OHMIM], (2-hydroxyethyl)trimethylammonium [Choline], tetraethylammonium [TEA], cetylpyridinium [C_16_Pyr] and trihexyltetradecylphosphonium [P_6,6,6,14_]. These cations were selected due to their low toxicity, except for [P_6,6,6,14_], which was chosen because it usually produces room temperature ionic liquids (RTILs). Cetylpyridinium chloride is also already used in some types of mouthwashes and toothpastes [53], although it is irritant in higher concentrations [54]. For control purposes in the biological activity studies, we also prepared the corresponding sodium and potassium salts of the hydrolyzed antibiotics by following the same synthetic procedure.

The isolation of these compounds was performed similarly to the previously reported ampicillin-based API-OSILs [18]. Briefly, the excess reactant was filtered-off after crystallization from acetonitrile/methanol (9:1), and this was followed by solvent evaporation. Table 1 shows the reaction yields, physical states and melting points of the prepared compounds. From the eleven synthesized API-OSILs, two were organic salts and nine were ionic liquids, including four RTILs.

The strict 1:1 cation–anion stoichiometry of the prepared API-OSILs, as well as the structural integrity of both components, was confirmed by ^1^H NMR spectroscopy. Further characterization was performed by ^13^C NMR and FTIR spectroscopies, as well as specific rotation, elemental analysis, and mass spectrometry. The ammonolysis of the β-lactam rings of the original APIs was confirmed by mass spectrometry. In the acquired electrospray ionization mass spectra (ESI-MS) spectra of all analyzed API-OSILs in the negative mode, the base peak corresponded to [M + 17]^−^ (*m/z*), which was consistent with the β-lactam ring opening with the consequent formation of an amide group and a secondary amine at the thiazolidine group.

### 2.2. Biological Activity

In the present study, all prepared compounds were tested against several sensitive and resistant Gram-positive and Gram-negative bacteria strains: *Staphylococcus aureus* ATCC 25923, *Escherichia coli* ATCC 25922, methicillin resistant *Staphylococcus aureus* (MRSA ATCC 43300) and *E. coli* CTX M2 and CTX M9.

The minimum inhibitory concentration (MIC) values were determined from three assays in triplicate by the broth micro dilution method in a 96-well microtiter plate by using tryptic soy broth (TSB) and adapted methodology from the Clinical Laboratory Standard Institute (CLSI) [62]. The strains were grown individually on tryptic soy agar for 24 h at 37 °C prior to each antibacterial test. Preceding MIC determination, each inoculum density was adjusted in TSB to 0.5 McFarland standards with a photometric device [62]. This resulted in a suspension that contained approximately 1 × 10^8^ to 2 × 10^8^ colony forming units (CFUmL^−1^) for *E. coli* ATCC25922^®^ [60]. A similar approach was used for the other strains. Then, 0.5 μL of the suspension was added to each well to have 5000–25,000 CFUmL^−1^. Bacteria were exposed to API-OSIL concentrations of 5, 2.5, 0.5, 0.05, 0.005, 0.0005, and 0.00005 mM. All compounds were dissolved in water except for OSILs that contained [P_6,6,6,14_] and [C_16_Pyr] cations, which were diluted in 1% dimethyl sulfoxide (DMSO). Their activity was determined in aqueous media, and the results of their activity were compared with bacteria that had been grown in TSB broth in the presence of 1% DMSO. The MIC for each compound was recorded as lowest molar concentration, showing no turbidity after 24 h of incubation at 37 °C [63,64]. The presence of turbidity was an indication of microbial growth, and the corresponding concentration of antibacterial agent was considered ineffective. The pharmacological activity of the prepared compound was then compared to the parent commercial API in terms of the relative decrease of inhibitory concentration (RDIC) as described earlier [16]. Herein, the RDIC value was calculated by dividing the MIC of the commercial API (penicillin G potassium salt or amoxicillin trihydrate) by the MIC of the corresponding synthesized compound.

### 2.3. Studies on Sensitive Bacteria

Table 2 shows data from the bioactivity study of the prepared compounds on *S. aureus* and *E. coli* sensitive strains.

The data gathered in Table 2 show that, on a first approach, the hydrolyzed salts of both Amx and Pen lost all antimicrobial activity against the tested sensitive strains. In fact, this was true for the vast majority of prepared OSILs. It has been a consistently and firmly established belief that the antimicrobial activity of β-lactam antibiotics relies on the sacrificial action of these functional groups [65] on transpeptidases that are responsible for the last cross-linking step of peptidoglycan synthesis in the bacterial cell wall. For that purpose, the transpeptidase active site (Ser residues in the case of D, and D- and Cys residues in the case of L,D-transpeptidases) nucleophilically attacks the nucleophilicity the carbonyl of the β-lactam ring, resulting in its opening of the ring and the irreversible formation of a covalent and stable acyl-enzyme complex [66,67]. However, this premise is widely accepted but poorly demonstrated. Recently, it was shown that even the acylation reactions of Cys residue of L,D-transpeptidases can be reversible, thus leading to limited antibacterial activity [68].

The same result was obtained for the majority of the prepared salts, irrespective of the cation polarity, resembling an ion trapping effect [69], i.e., ionic compounds such as [*seco*-Amx] and [*seco*-Pen] and their conjugated acids are subject to a variety of processes, such as dissociation, electrical interactions with organic matter, and changes in their partitioning in hydrophobic/hydrophilic media. These processes depend on pH, ionic strength, their polarity, and pK_a_, and they ultimately lead to their accumulation in certain zones of the bacterial cell, i.e., the ion-trap effect. On the other hand, highly polar cations are more prone to closely interact with them in anionic form, anchoring them in the polar solution. This effect has also been seen by us with ampicillin-based API-OSILs against sensitive Gram-positive and Gram-negative species [16] and seem to adversely affect the activity of those compounds. In the case of highly hydrophobic cations, the micelles of API-OSILs may be formed, thus reducing their antimicrobial activity [70].

The only observed exceptions were [C_2_OHMIM][*seco*-Amx], [C_2_OHMIM][*seco*-Pen], and [TEA][*seco*-Pen]. While the first one showed no advantage over Amx (RDIC = 1) against *S. aureus*, the second and third *seco*-Pen OSILs recorded RDICs of 100 (*S. aureus*) and 10 (*E. coli*), respectively.

While inactive in the Cl^−^ salt form, the [C_2_OHMIM]^+^ cation was the only selected cationic entity that allowed for the enhancement of the antimicrobial activity of both *seco*-Amx and *seco*-Pen against this sensitive *S. aureus* strain. These results most probably come from specific intermolecular interactions between the cation and the anion, thereby enabling the deactivating interactions of crucial PBPs. PBP:API-OSILs interaction studies will be conducted in the future and published accordingly.

In the case of the sensitive Gram-negative *E. coli* strain, its outer membrane can hinder drug delivery, as proposed above. In fact, the activity of [*seco*-Amx] and [*seco*-Pen] towards this strain did not seem to be enhanced by the combination with [C_2_OHMIM] or with any of the other cations, with the exception of [TEA] (RDIC = 10). In truth, [TEA][*seco*-Pen] was found to be ten times more effective than the parent K[Pen]. These results are probably related with augmented hindrance of the porin entrance and/or uncompetitive phase transfer delivery through the outer membrane in comparison with the free antibiotic.

Similar results were also recorded in a past study regarding ampicillin-based API-ILs with sensitive Gram-positive and Gram-negative bacteria [16]. Recent results in the literature regarding API-OSILs as antibiotics against sensitive bacteria have referred to discrete interactions with the bacteria cell wall or membrane [71,72,73,74,75,76,77,78,79]. In particular, pyridyl cationic-modified benzylidene cyclopentanone photosensitizers (PSs) that were developed by Wu et al. [77] showed that Gram-positive bacteria are more sensitive than Gram-negative bacteria to photodynamic therapy because their walls are more porous and all types of PSs can readily diffuse through them. In contrast, Gram-negative bacteria possess an additional negatively charged outer layer that serves as a permeability barrier, so neutral and anionic PSs often fail to effectively inactivate Gram-negative bacteria, while cationic PSs can still strongly bind to their outer membrane and damage their integrity. In addition, antimicrobial studies that were supported by FTIR spectroscopy experiments revealed that nalidixic acid salts with particular ammonium cations exhibit enhanced antimicrobial activity against six different Gram-negative *Salmonella* species and two nalidixic acid-resistant *S. typhimurium* strains by displaying different modes of action towards proteins, carbohydrates, and lipids within the cell membrane [78]. One final example concerning FTIR bioassays revealed that hydrophobic *N*-alkyltropinium bromide surfactants preferably interact with the fatty acids and amide groups within the cell envelope of Gram-negative *E. coli* and with the peptidoglycan multilayer of the Gram-positive *Listeria innocua* cells [79]. The interaction of the ILs based on penicillin and amoxicillin, as well as [*seco*-Pen] and [*seco*-Amx] anions with the cell membrane of Gram-positive and Gram-negative bacteria strains, will be studied soon and published accordingly.

### 2.4. Studies on Resistant Bacteria

The prepared OSILs from hydrolyzed amoxicillin and penicillin antibiotics were also studied against resistant Gram-negative *E. coli* strains CTX M9 and CTX M2, as well as the methicillin-resistant *S. aureus* ATCC 43,300 (see Table 3).

As expected, parent antibiotics (Amx and [K][Pen]), as well as the sodium and potassium salts of their ammonia hydrolysates, were found to be inactive against all tested resistant bacteria strains. For *E. coli*, five OSILs that contained the [*seco*-Amx] anion and three that contained the [*seco*-Pen] anion showed increased activity (RDIC values between >5 and >100) against the parent antimicrobials. The highest activity (RDIC > 100) was recorded for [C_16_Pyr][*seco*-Amx] against both resistant *E. coli* strains, while [P_6,6,6,14_][*seco*-Amx] and [Choline][*seco*-Amx] were selective towards only one of the strains—CTX M9 and CTX M2, respectively.

These results were somewhat similar to those regarding ampicillin-based API-OSILs against resistant Gram-negative *E. coli* species that were previously described by us [16], where [C_16_Pyr][Amp] and [P_6,6,6,14_][Amp] showed the highest antimicrobial activities of all compounds tested. Therein we suggested that the drug delivery of the APIs is enhanced in some resistant *E. coli* strains by the lipophilic counter-ions through the permeation of the outer layer. This postulation is supported by results from other authors. More specifically, Vincent et al. [80] and Langgartner, J. et al. [81] similarly demonstrated that transport across biological membranes bearing a highly polar anionic framework can be facilitated if the APIs are paired with lipophilic ammonium ions that act as phase transfer agents. Additionally, Rogers et al. [82] recently demonstrated on synthetic membrane models that API-OSILs that contain lipophilic cations, preferably with established hydrogen bonds, exhibit an increased membrane transport as compared to API-OSILs with weaker electrostatic interactions or even traditional halide or metal salts. Finally, it is important to note that both hydrophobic and hydrophilic ionic liquids have been recently studied as penetration enhancers [16,20,83,84]. Various nanocarriers can serve as antimicrobial enhancers because they incorporate into lipid membranes or cell walls, leading to membrane or wall disruptions in both Gram-positive and Gram-negative bacteria strains [77,85,86,87,88,89,90,91], therefore increasing the drug’s efficiency.

The activity of prepared OSILs against the Gram-positive MRSA ATCC 43,300 strain seems even more peculiar. IC_50_ values as low as 5 and 50 µM, respectively, were obtained with [C_16_Pyr][*seco*-Amx] and [C_16_Pyr][*seco*-Pen], which corresponded to RDICs higher than 1000 and 100, respectively. The only other measurable value was obtained with [Choline][*seco*-Pen] (RDIC > 5). In the former cases, the contribution from the [C_16_Pyr] cation was unquestionable. Literature data [92] have shown that the MIC for [C_16_Pyr][Cl] is five times higher in the methicillin-resistant than in methicillin-sensitive *S. aureus* strains, suggesting that the contribution of this cation to the antibacterial activity of these particular API-OSILs [92] is particularly significant as opposed to the parent antibiotics or their hydrolyzed analogues (see Table 3). In other words, the amplified activity of [C_16_Pyr][*seco*-Amx] and [C_16_Pyr][*seco*-Pen] can only be achieved due to a synergic effect of both ionic species. Such a strong influence of [C_16_Pyr]^+^ seems quite in contrast with the unspecific activity of so-called enhancers (such as [C_2_OHMIM]^+^ for sensitive *S. aureus* above) and prompt us to consider it as a β-lactam antibiotic potentiator [63,72]. Similar results from the literature have shown that berberine in the presence of ampicillin and oxacillin markedly lowers their MICs against MRSA despite berberine alone exhibiting no bactericidal activity. Later, it was shown that, in fact, berberine affected MRSA biofilm development and the dissemination of biofilm-associated infections [93,94]. This effect is most likely related with the formation of salt bridges at an allosteric site of the PBP2a in MRSA ATCC 43300 [92]. Curiously, similar interactions were also found for another alkylpyridinium compound, namely the antibiotic ceftaroline [95]. Thus, an analogous ionic allosteric effect at PBP2a may be occurring with the described [C_16_Pyr]-based OSILs. Further studies, namely molecular dynamic and docking simulations, must be performed in the future to confirm this assumption. Regardless of the mechanism, the pronounced increase in RDIC values of the prepared API-OSILs, particularly against resistant species, seems very interesting for a potential drug combination strategy. In spite of some controversy, the combination of antimicrobials with non-active compounds may provide a quite promising strategy to address the widespread emergence of antibiotic-resistant bacteria strains [96,97,98].

## 3. Conclusions

The present work highlights that organic salts and ionic liquids that contain ammonia hydrolysates of amoxicillin and penicillin G (*seco*-Amx and *seco*-Pen), in particular [C_2_OHMIM][*seco*-Amx], [C_2_OHMIM][*seco*-Pen], [TEA][*seco*-Pen], [C_16_Pyr][*seco*-Amx], abd [Choline][*seco*-Amx], [C_16_Pyr][*seco*-Pen] display a very strong antibacterial effect on sensitive and resistant *E. coli* and MRSA strains, respectively. The gathered data suggest that the adequate ionic pairing of such hydrolyzed antimicrobials with an ion-pair effect is vital to enhance or promote antibiotic activity, with possible alterations in their mechanism of action according to the selected counter-ion. In particular, the hydrophobic combination [C_16_Pyr][*seco*-Amx] demonstrated the highest efficiency towards resistant bacteria strains, with particular emphasis to MRSA ATCC 43300. The combination of [C_16_Pyr] with [*seco*-Pen] was also very effective against the latter. These results are clearly promising and point towards a beneficial effect on the drug delivery of the modified APIs when combined with hydrophobic organic cations. In this way, the antimicrobial resistance to these standard β-lactam antibiotics can be drastically reduced in vitro.

Our results also show that future developments of novel APIs-OSILs must not only focus on the toxicity and hydrophobicity of the counter ion—they must also look at the outcome. More specifically, [P_6,6,6,14_][*seco*-Amp] follows the trend of [C_16_Pyr][Amp] on *E. coli* resistant strains, which suggests that there may be other factors at stake to be considered.

The virtually limitless number of ionic pairs that can be assembled as API-OSILs makes this area of research particularly interesting and potentially thriving. In addition, the straightforward synthetic procedure adds virtually no barriers to its future industrial up-scaling and will thus eventually lead to an effective combination therapy model to tackle the ever-emerging bacterial resistance towards antibiotics.

## 4. Experimental Section

### 4.1. Synthesis

Commercially available reagents were purchased from Aldrich, BDH—the Frilabo and Solchemar laboratory reagents were used as received. The solvents were from Valente & Ribeiro and distilled before use. Whenever necessary, the solvents were dried by standard procedures, distilled under nitrogen and stored over molecular sieves.

The basic anion-exchange resin Amberlite IRA-400-OH (ion-exchange capacity 1.4 eq.mL^−1^) and Amberlyst A-26 resins were purchased from Supelco. ^1^H and ^13^C-NMR spectra in (CD_3_)_2_SO, CD_3_OD or D_2_O (from Euriso-Top) were recorded on a Bruker AMX400 spectrometer at room temperature unless specified otherwise. To perform NMR, 5 mm borosilicate tubes were used, and the sample concentration was, approximately, 7 mg/mL for ^1^H-NMR and 37 mg/mL for ^13^C-NMR. Chemical shifts are reported downfield in parts per million (ppm).

ESI-MS were acquired with an API-ION TRAP(PO03MS), ITQB, Oeiras, Portugal, operating in both positive- and negative ion modes and equipped with a Z-spray source. Source and desolvation temperatures were 80 and 100 °C, respectively. The ionic liquid solutions in methanol at concentrations ∼10–4 mol dm^−3^ were introduced at a 10 µl min^−1^ flow rate. The capillary and the cone voltage were 2600 and 25 V, respectively. Nitrogen was used as a nebulization gas and argon was used as a collision gas. ESI-MS-MS were acquired by selecting the precursor ion with the quadrupole and then performing collisions with argon at energies from 3 to 20 eV in the hexapole.

**Figure 2:**
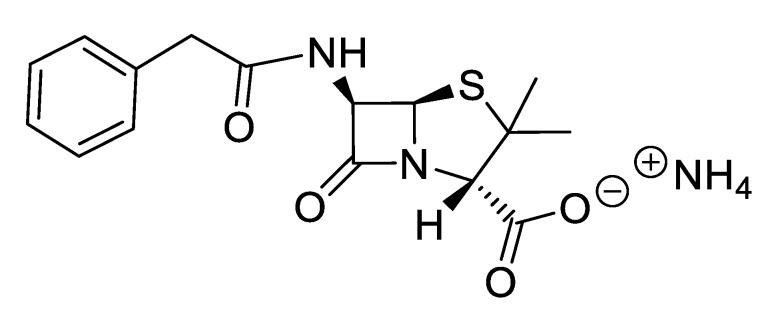
[NH_4_][Pen].

**Figure 3:**
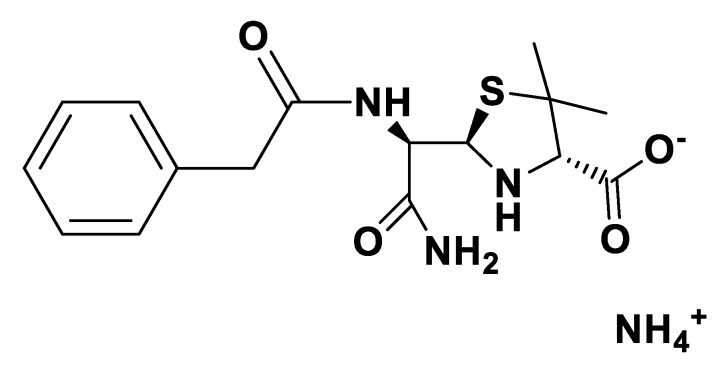
[NH_4_][*seco*-Pen].

**Figure 4:**
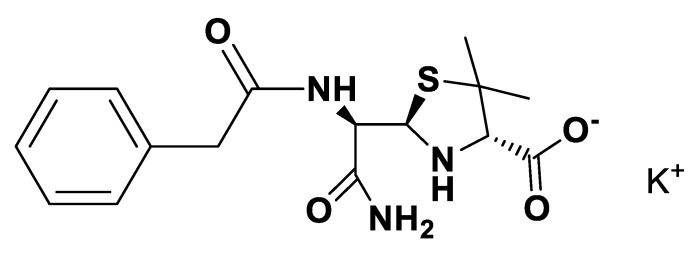
[K][*seco*-Pen].

**Figure 5:**
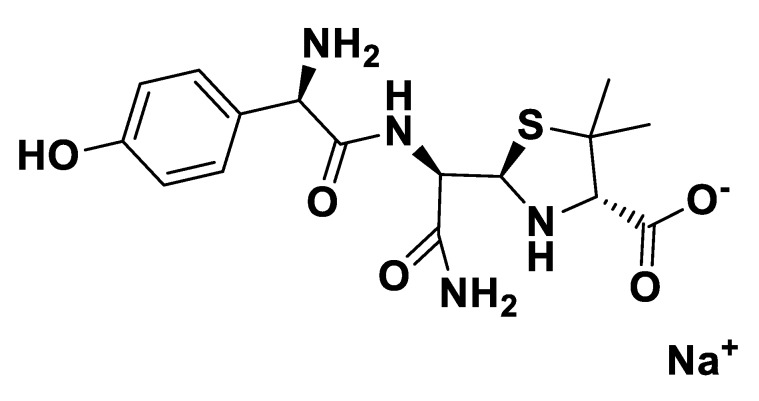
[Na][*seco*-Amx].

**Figure 6:**
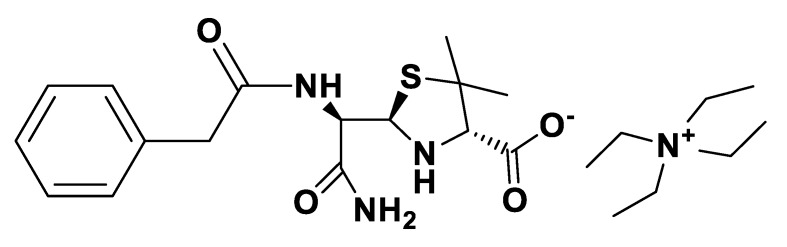
[TEA][*seco*-Pen].

**Figure 7:**
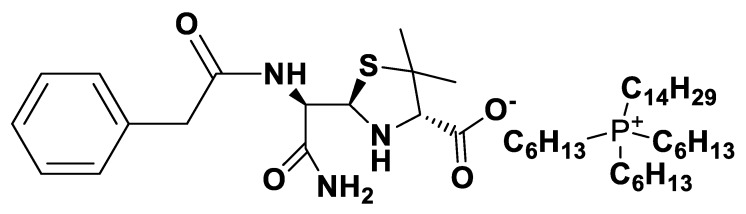
[P_6,6,6,14_][*seco*-Pen].

**Figure 8:**
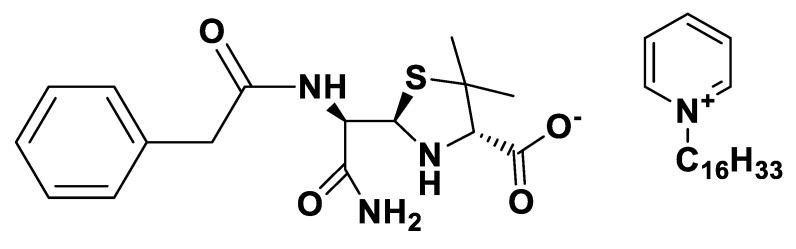
[C_16_Pyr][*seco*-Pen].

**Figure 9:**
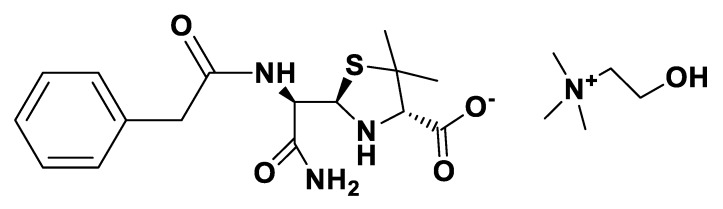
[Choline][*seco*-Pen].

**Figure 10:**
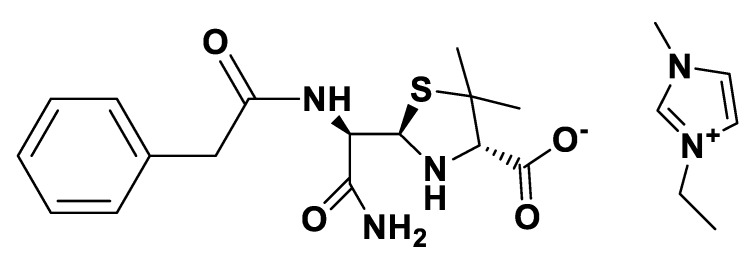
[EMIM][*seco*-Pen].

**Figure 11:**
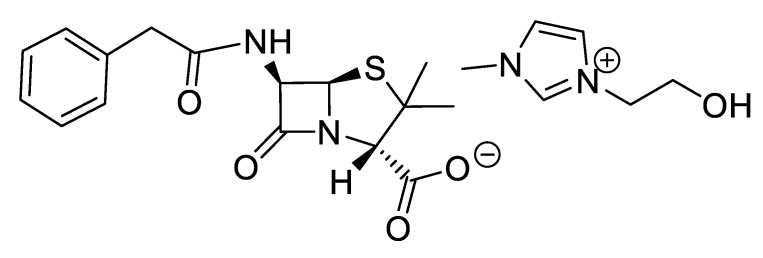
[C_2_OHMIM][*seco*-Pen].

**Figure 12:**
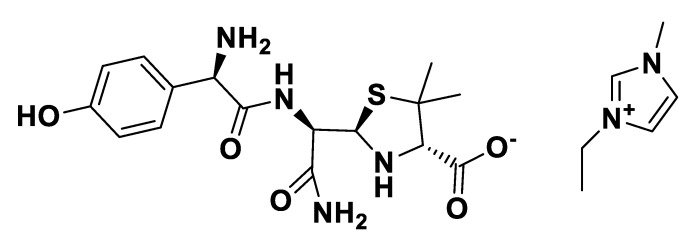
[EMIM][*seco*-Amx].

**Figure 13:**
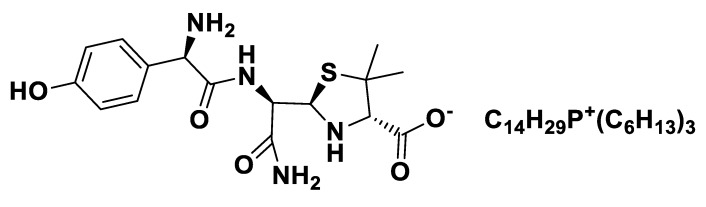
[P_6,6,6,14_][*seco*-Amx].

**Figure 14:**
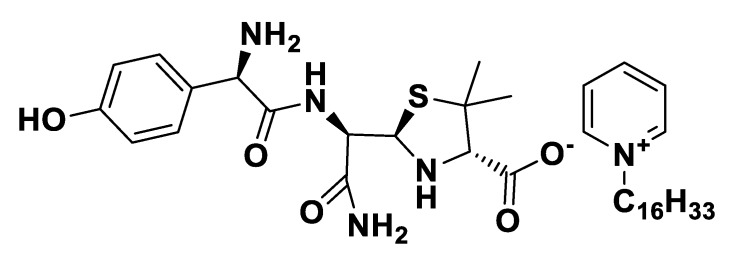
[C_16_Pyr][*seco*-Amx].

**Figure 15:**
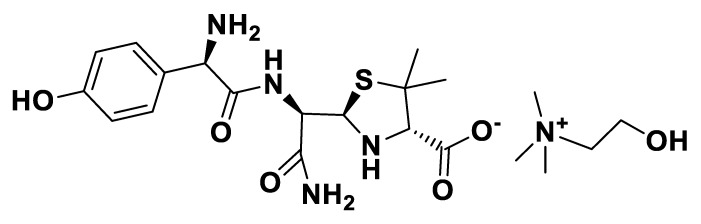
[choline][*seco*-Amx].

**Figure 16:**
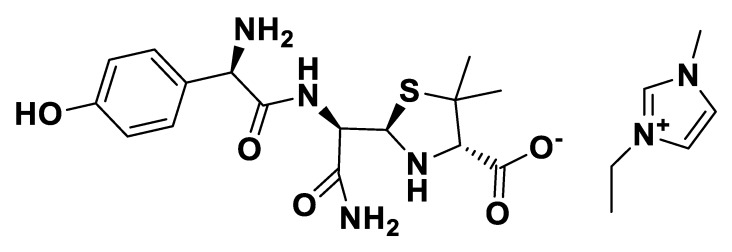
[C_2_OHMIM][*seco*-Amx].

IR spectra were measured on a Perkin Elmer 683 by using KBr sample disks. Optical rotations were recorded on a Perkin Elmer 241MC. The melting temperature (mT) was determined with a melting point apparatus (Stuart Scientific). The elemental analysis experiments were performed in a CHNS Series Thermo Finnigan-CE Instruments Flash EA 1112 under standard conditions (T combustion reactor 900 °C, T GC column furnace 65 °C, multiseparation SS GC column, He_2_ flow 130 mL/min, O_2_ flow 250 mL/min). The penicillin G potassium was transformed in penicillin G ammonium by the adaptation of the method of Salivar, C. J. et al. [63]. Figure 2, Figure 3, Figure 4, Figure 5, Figure 6, Figure 7, Figure 8, Figure 9, Figure 10, Figure 11, Figure 12, Figure 13, Figure 14, Figure 15 and Figure 16 illustrate the chemical structures of all prepared OSILs based APIs.

#### 4.1.1. Synthesis of seco-Pen-Based OSILs

The preparation of ammonium (2S,5R,6R)-3,3-dimethyl-7-oxo-6-(2-phenylacetamido)-4-thia-1-azabicyclo [3.2.0]heptane-2-carboxylate [NH_4_][Pen] was done as indicated by Salivar et al method.

The penicillin G potassium was transformed in penicillin G ammonium following the method of Salivar, C. J. et al. [63] before the reaction with hydroxide reactants.

##### Preparation of [NH_4_][*seco*-Pen]

Potassium penicillin (1 g; 2.6 mmol) was dissolved in 15 mL of a 1.0 M aqueous ammonium solution. To the solution was added Amberlyst A-26 resin (5 eq.) that was previously stirred in a 2.0 M aqueous ammonium solution for 1 hour. The reaction mixture was stirred at room temperature for an additional 1 h. The resin was filtered off, and the was solvent evaporated to provide the desired product as a grey solid (0.828 g; 83%); ^1^H NMR (400 MHz, D_2_O) δ(ppm): 7.33–7.43 (m, 5H); 4.99 (d, *J* = 8 Hz, 1H); 4.81(s, 1H); 4.41 (d, *J* = 7.6 Hz, 1H); 3,69 (s, 2H); 3.50 (s, 1H); 1.56 (s, 3H); 1.25 (s, 3H); ^13^C NMR (100 MHz, D_2_O) δ(ppm): 177.55; 177.40; 176.65; 137.54; 132.14; 131.79; 130.22; 77.42; 67.24; 61.68; 60.93; 44.87; 29.56; 29.30.IR: υ = 3171; 2964; 1644; 1571; 1494; 1454; 1381; 1188; 1130; 1073; 1032; 784; 692; 502. Elemental analysis calculated for C_16_H_24_N_4_O_4_S⋅0.8H_2_O: C 50.19; H 6.74; N 14.63. Found: C 50.11; H 6.67; N 14.97.

##### Preparation of [K][*seco*-Pen]

Potassium penicillin (0.137g; 0.36 mmol) was dissolved in a 1.0 M aqueous ammonium solution. The mixture was stirred at room temperature for 4 h. The solvent was evaporated to provide the desired product as a yellow solid (0.139 g; 97%); m.p. 193–195 °C; ^1^H NMR (400 MHz, D_2_O) δ(ppm): 7.34–7.44 (m, 5H); 4.98 (d, *J* = 7.6 Hz, 1H); 4.83 (t, *J* = 1.6 Hz, 1H); 4.39 (d, *J* = 8 Hz, 1H); 3.70 (s, 2H); 3.47 (s, 1H); 1.57(s, 3H); 1.25 (s, 3H); ^13^C NMR (100 MHz, D_2_O) δ(ppm): 177.82; 177.44; 176.79; 137.56; 131.77; 132.13; 130.19; 77.58; 67.42; 62.05; 61.20; 44.85; 29.49; 29.37; IR: υ = 3288; 2969; 2925; 1648; 1582; 1496; 1454; 1382; 1364; 1257; 1128; 877; 791; 694; 502. Elemental analysis calculated for C_16_H_20_KN_4_O_5_S⋅3H_2_O: C 44.22; H 5.80; N 9.67. Found: C 44.45; H 5.54; N 9.70.

##### Preparation of [Na][*seco*-Amx]

Amoxicillin (0.127 g; 0.3 mmol) was dissolved in a 1.0M aqueous ammonium solution. After 20 min, NaOH (0.012 g; 0.3 mmol) was added, and the mixture was stirred at room temperature for 4 h. The solvent was evaporated to provide the desired product as a yellow solid (0.127 g; 96%); m.p. 137–139 °C; ^1^H NMR (400 MHz, D_2_O) δ(ppm): 7.30 (d, *J* = 8 Hz, 2H); 6.89 (d, *J* = 8.4 Hz, 2H); 4.35 (d, *J* = 7.2 Hz, 1H); 3.29 (s, 1H); 1.38 (s, 3H); 1.17 (s, 3H); ^13^C NMR (100 MHz, D_2_O) δ(ppm): 177.77; 177.05; 176.60; 158.88; 131.63; 118.81; 77.61; 67.33; 61.91; 60.77; 60.23; 29.05; 28.85; IR: υ = 3277; 2969; 2919; 1648; 1575; 1510; 1433; 1383; 1322; 1245; 1173; 1127; 981; 865; 817; 780. Elemental analysis calculated for C_16_H_21_N_4_NaO_5_S⋅3H_2_O: C 41.92; H 5.94; N 12.22. Found: C 41.87; H 6.00; N 11.99.

##### Preparation of [TEA][*seco*-Pen]

Tetraethylammonium bromide (0.420 g; 2.00 mmol) was dissolved in methanol and passed through Amberlite IRA-400-OH an ion-exchange column [18,99] (5 eq., flux rate 0.133 mLmL^−1^min^−1^ = 8 BVh^−1^). Then, the tetraethylammonium hydroxide solution that was formed was slowly added to the ammonium penicillin G (0.751 g; 2.14 mmol) that was dissolved in the 1.0 M aqueous ammonium solution (50 mgmL^−1^). The reaction mixture was stirred at room temperature for 1 h. After solvent evaporation, the residue was dissolved in a 20 mL solution (methanol/acetonitrile 1:9) [18,99] and left refrigerated overnight (4 °C) [18,99] to induce the precipitation of the excess of reagents. When the reagent crystals were filtered out, the solution was evaporated, and the rest was dried in a vacuum for 24 h to provide the desired product as a yellow viscous liquid (0.856 g; 90%). [α]_D_^25^ = 104.0 ± 6.1 (c = 2 mgmL^−1^ in methanol), ^1^H-NMR (400.13 MHz, CD_3_OD) δ = 7.31–7.22 (m, 5H), 5.46 (s, 1H), 4.17 (s, 1H) 3.70 (s, 1H), 3.63-3.58 (m, 2H), 3.51 (bs, 1H), 3.27–3.26 (m, 8H), 1.63 (s, 3H), 1.55 (s, 3H), 1.30–1.24 (m, 12H); ^13^C-NMR (100.62 MHz, CD_3_OD) δ = 174.72, 174.37, 174.15, 140.88, 136.73, 130.31, 130.25, 129.63, 127.96, 75.73, 66.68, 60.19, 59.27, 53.29, 46.65, 43.83, 29.41, 28.67, 28.38, 27.66, 7.64 ppm; IR (KBr): υ = 3420, 2981, 2924, 2862, 1840, 1736, 1721, 1648, 1560, 1543, 1490, 1459, 1432, 1396, 1367, 1173, 1130, 1053, 1027, 1001, 785, 734, 696, 619, 539 cm^−1^; (ESI^+^) *m/z* calculated for C_8_H_20_N^+^: 130.1 found 130.0; (ESI^−^) *m/z* calculated for C_16_H_20_N_3_O_4_S^−^ 350.1 found 349.8.

##### Preparation of [P_6,6,6,14_][*seco*-Pen]

Trihexyl(tetradecyl)phosphonium chloride (1.000 g; 1.92 mmol) was dissolved in methanol and passed through an Amberlite IRA-400-OH [18,99] ion-exchange column (5 eq., flux rate 0.133 mLmL^−1^min^−1^ = 8 BVh^−1^). Then, the trihexyl(tetradecyl)phosphonium hydroxide solution that was formed was slowly added to ammonium penicillin G (0.853 g; 2.43 mmol) and dissolved in a 1.0 M aqueous ammonium solution (50 mgmL^−1^). The mixture was stirred at room temperature for 1 h. After solvent evaporation, the residue was dissolved in a 20 mL solution (methanol/acetonitrile 1:9) [18,99] and left refrigerated overnight (4 °C) [14] to induce the precipitation of the excess reagent. When the reagent crystals were filtered out, the solution was evaporated, and the rest was dried in a vacuum for 24 h to provide the desired product as a yellow viscous liquid (1.560 g; 97%). [α]_D_^25^ = 67.7 ± 3.0 (c = 2 mgmL^−1^ in methanol); ^1^H-NMR (400.13 MHz, CD_3_OD) δ = 7.34–7.22 (m, 5H), 4.97 (1H, d, *J* = 6.7 Hz), 4.34 (dd, 1H, *J* = 6.7 Hz), 3.60 (d, 2H, *J* = 8.2 Hz), 3.50 (s, 1H), 2.20 (m, 8H), 1.56–1.25 (m, 54H), 0.96–0.88 (m, 12H) ppm; ^13^C-NMR (100.62 MHz, CD_3_OD) δ = 175.30, 174.83, 173.92, 136.76, 130.37, 129.59, 127.87, 76.81, 66.78, 60.14, 54.86, 43.68, 33.11, 32.19, 31.92, 31.84, 30.80, 30.51, 30.45, 29.91, 27.88, 23.77, 23.49, 22.36, 19.53, 19.05, 14.51, 14.38 ppm; IR (KBr): ν = 3308, 3028, 2951, 2923, 2853, 1737, 1669, 1607, 1536, 1496, 1456, 1418, 1379, 1262, 1201, 1113, 1031, 986, 860, 810, 761, 722, 694, 617, 454, 439, 424 cm^−1^; (ESI^+^) *m/z* calculated for C_32_H_68_P^+^: 483.4 found 483.8; (ESI^−^) *m/z* calculated for C_16_H_20_N_3_O_4_S^−^ 350.1, found 349.9.

##### Preparation of [C_16_Pyr][*seco*-Pen]

##### Procedure I

Cetylpyridinium chloride (0.822 g; 2.30 mmol) was dissolved in methanol and passed through an Amberlite IRA-400-OH ion-exchange column [18,99] (5 eq., flux rate 0.133 mLmL^−1^min^−1^). Then, the cetylpyridinium hydroxide solution that was formed was slowly added to ammonium penicillin G (0.973 g; 2.77 mmol) that was dissolved in a 1.0 M aqueous ammonium solution (50 mgmL^−1^). The mixture was stirred at room temperature for 1 h. After solvent evaporation, the residue was dissolved in a 20 mL solution (methanol/acetonitrile 1:9) [18,99] and left refrigerated overnight (4 °C) [18,99] to induce the precipitation of the excess reagent. When the reagent crystals were filtered out, the solution was evaporated, and the rest was dried in a vacuum for 24 h to provide the desired product as a yellow solid (1.332 g; 89%). m.p. 76–78 °C; [α]_D_^25^ = 47.3 ± 3.6 (c = 2 mgmL^−1^ in methanol); ^1^H-NMR (400.13 MHz, CD_3_OD) δ = 9.01 (d, 2H, *J* = 5.7 Hz), 8.59 (t, 1H, *J* = 7.8 Hz), 8.12 (t, 2H, *J* = 6.8 Hz), 7.33–7.21 (m, 5H), 4.95 (d, 1H, *J* = 7.1 Hz), 4.63 (t, 2H, *J* = 7.5Hz,), 4.35 (d, 1H, *J* = 7.0 Hz), 3.60 (2H, d, *J* = 7.5 Hz), 3.50 (s, 1H), 1.56 (m, 3H), 1.42-1.09 (m, 31H), 0.90 (t, 3H, *J* = 6.6 Hz) ppm; ^13^C-NMR (100.62 MHz, CD_3_OD) δ = 175.16, 174.78, 173.98, 146.87, 146.00, 136.73, 130.40, 129.80, 129.60, 127.93, 76.34, 66.64, 63,15, 60.14, 43.67, 33.12, 32.55, 30.81, 30.67, 30.17, 27.86, 27.24, 23.78, 14.49 ppm; IR (KBr): δ = 3041, 3059, 2914, 2848, 1739, 1658, 167, 1601, 1542, 1528, 1508, 1487, 1472, 1397, 1368, 1322, 1270, 1209, 1177, 1128, 1078, 1032, 987, 960, 926, 818, 777, 716, 686, 619, 574, 475 cm^−1^; (ESI^+^) *m/z* calculated for C_21_H_38_N^+^: 304.3 found 304.2; (ESI^−^) *m/z* calculated for C_16_H_20_N_3_O_4_S^−^ 350.1, found 349.9.

##### Procedure II

Cetylpyridinium chloride (0.145g; 0.43 mmol) was dissolved in methanol and was added Amberlyst A-26 (3 eq.) The mixture was stirred for 1 h at room temperature. Then, the cetylpyridinium hydroxide solution that was formed was slowly added to [NH_4_][*seco*-Pen] (0.150 g; 0.43 mmol) that was dissolved in a 2.0 M aqueous ammonium solution, and the mixture was stirred at room temperature for 1 h. The solvent was evaporated to p rovide the desired product as a white solid (0.263 g; 94%).

##### Preparation of [Choline][*seco*-Pen]

(2-hydroxyethyl)trimethylammonium chloride (0.277 g; 1.99 mmol) was dissolved in methanol and passed through an Amberlite IRA-400-OH ion-exchange column [18,99] (5 eq., flux rate 0.133 mLmL^−1^min^−1^ = 8 BVh^−1^). Then, the hydroxide solution that was formed was slowly added to ammonium penicillin G (0.848 g; 2.41 mmol) that was dissolved in a 1.0 M aqueous ammonium solution (50 mgmL^−1^). The mixture was stirred at room temperature for 1 h. After solvent evaporation, the residue was dissolved in a 20 mL solution (methanol/acetonitrile 1:9) [18,99] and left refrigerated overnight (4 °C) [18,99] to induce the precipitation of the excess reagent. When the reagent crystals were filtered out, the solution was evaporated, and the rest was dried in a vacuum for 24 h to provide the desired product as a yellow solid (0.856 g; 95%). m.p. 69–71 °C; [α]_D_^25^ = 47.3 ± 3.6 (c = 2 mgmL^−1^ in methanol); ^1^H-NMR (400.13 MHz, CD_3_OD) δ = 7.33–7.29 (m, 5H), 4.95 (d, 1H, *J*_1_ = 7.0 Hz), 4.35 (d, 1H, *J*_1_ = 7.0 Hz), 4.02–3.98 (m, 2H), 3.66–3.56 (m, 2H), 3.50-3.47 (m, 3H), 3.20 (s, 9H) 1.56 (s, 3H), 1.25 (s, 3H) ppm; ^13^C-NMR (100.62 MHz, CD_3_OD) δ = 174.72, 174.37, 174.15, 140.88, 136.73, 130.31, 130.25, 129.63, 127.96, 75.73, 69.06, 66.67, 60.20, 60.04, 59.53, 57.10, 46.65, 27.78, 27.47 ppm; IR (KBr): υ = 3468, 3074, 2966, 1652, 1496, 1479, 1461, 1396, 1356, 1279, 1204, 1131, 1083, 1054, 1010, 955, 887, 867, 796, 734, 702, 670, 620, 540, 476 cm^−1^; (ESI^+^) *m/z* calculated for C_5_H_14_NO^+^: 104.1, found 104.1; (ESI^−^) *m/z* calculated for C_16_H_20_N_3_O_4_S^−^ 350.1, found 349.9.

##### Preparation of [EMIM][*seco*-Pen]

1-ethyl-3-methylimidazolium bromide (0.578 g; 3.03 mmol) was dissolved in methanol and passed through an Amberlite IRA-400-OH ion-exchange column [18,99] (5 eq., flux rate 0.133 mLmL^−1^min^−1^ = 8 BVh^−1^). Then, the hydroxide solution that was formed was slowly added to ammonium penicillin G (1.11 g; 3.16 mmol) that was dissolved in an aqueous 1.0 M ammonium solution (50 mgmL^−1^). The mixture was stirred at room temperature for 1 h. After solvent evaporation, the residue was dissolved in a 20 mL solution (methanol/acetonitrile 1:9) [18,99] and left refrigerated overnight (4 °C) [18,99] to induce the precipitation of the excess reagent. When the reagent crystals were filtered out, the solution was evaporated, and the rest was dried in a vacuum for 24 h to provide the desired product as a colorless viscous liquid (1.136 g; 81%). [α]_D_^25^ = 89.0 ± 7.0 (c = 2 mgmL^−1^ in methanol); ^1^H-NMR (400.13 MHz, CD_3_OD) δ = 8.99 (bs, 1H), 7.63 (s, 1H) 7.53 (s, 1H), 7.34–7.20 (m, 5H), 4.95 (d, 1H, *J* = 7.0 Hz), 4.35 (d, 1H, *J* = 7.0 Hz), 4.24 (q, 2H, *J* = 7.3 Hz), 3.92 (s, 3H), 3.60 (d, 2H, *J* = 7.2 Hz), 3.50 (s, 1H), 1,60–1.50 (m, 6H), 1.25 (s, 3H) ppm; ^13^C-NMR (100.62 MHz, CD_3_OD) δ = 175.06, 174.83, 173.99, 136.80, 130.65, 130.39, 129.60, 127.90, 124.94, 123.25, 76.51, 60.13, 60.18, 60.11, 59.54, 46.02, 43.66, 36.51, 27.81, 27.65, 15.61 pm; IR (KBr): υ = 3468, 3368, 2970, 1660, 1540, 1501, 1456, 1395, 1456, 1395, 1354, 1300, 1258, 1169, 1131, 1301, 965, 918, 862, 828, 729, 700, 651, 620, 545 cm^−1^; (ESI^+^) *m/z* calculated for C_6_H_11_N_2_^+^: 111.1, found 111.0; (ESI^−^) *m/z* calculated for C_16_H_20_N_3_O_4_S^−^ 350.1, found 349.9.

##### Preparation of [C_2_OHMIM][*seco*-Pen]

3-(2-hydroxyethyl)-1-methylimidazolium chloride (0.328 g; 2.03 mmol) was dissolved in methanol and passed through an Amberlite IRA-400(OH) ion-exchange column [14,41] (5 eq., flux rate 0.133 mLmL^−1^min^−1^ = 8 BVh^−1^). Then, the hydroxide solution that was formed was slowly added to ammonium penicillin G (0.754 g; 2.14 mmol) that was dissolved in a 1.0 M aqueous ammonium solution (50 mgmL^−1^). The mixture was stirred at room temperature for 1 h. After solvent evaporation, the residue was dissolved in a 20 mL solution (methanol/acetonitrile 1:9) [18,99] and left refrigerated overnight (4 °C) [18,99] to induce the precipitation of the excess reagent. When the reagent crystals were filtered out, the solution was evaporated, and the rest was dried in a vacuum for 24 h to provide the desired product as a yellow solid (0.799 g; 83%). m.p. 48–50 °C; [α]_D_^25^ = 41.3 ± 6.0 (c = 2 mgmL^−1^ in methanol); ^1^H-NMR (400.13 MHz, CD_3_OD) δ = 8.98, (s, 1H), 7.61 (s, 1H), 7.54 (s, 1H), 7.33–7.21 (m, 5H), 4.94 (d, 1H, *J* = 7.1 Hz), 4.36 (d, 1H, *J* = 7.1 Hz), 4.29 (t, 2H, *J* = 4.9 Hz), 3.92, (s, 3H), 3.86 (t, 2H, *J* = 4.9 Hz), 3.59 (d, 2H, *J* = 7.1 Hz), 3.50 (s, 1H), 1.55 (s, 3H), 1.24 (s, 3H) ppm; ^13^C-NMR (100.62 MHz, CD_3_OD) δ = 175.04, 174.82, 174.00, 136.78, 130.63, 130.51, 130.40, 129.61, 129.85, 129.61, 127.91, 124.68, 124.00, 76.50, 66.76, 61.10, 60.12, 59.53, 53.26, 43.65, 36.45, 27.84 ppm; IR (KBr): υ = 3418, 2965, 2931, 2108, 1644, 1585, 1499, 1455, 1398, 1356, 1260, 1167, 1127, 1076, 1034, 879, 798, 734, 704, 668, 619, 464, 445, 432, 424 cm^−1^; (ESI^+^) *m/z* calculated for C_6_H_11_N_2_O^+^: 127.2, found 127.0; (ESI^−^) *m/z* calculated for C_16_H_20_N_3_O_4_S^−^ 350.1, found 349.9.

#### 4.1.2. Synthesis of seco-Amx-Based OSILs

##### Preparation of [EMIM][*seco*-Amx]

1-ethyl-3-methylimidazolium chloride (0.385 g; 2.01 mmol) was dissolved in methanol and passed through an Amberlite IRA-400-OH ion-exchange column [18,99] (5 eq., flux rate 0.133 mLmL^−1^min^−1^ = 8 BVh^−1^). Then, the hydroxide solution that was formed was slowly added to amoxicillin trihydrate (0.930 g; 2.22 mmol) that was dissolved in an aqueous 1.0 M ammonium solution (50 mgmL^−1^). The mixture was stirred at room temperature for 1 h. After solvent evaporation, the residue was dissolved in a 20 mL solution (methanol/acetonitrile 1:9) [18,99] and left refrigerated overnight (4 °C) [18,99] to induce the precipitation of the excess reagent. When the reagent crystals were filtered out, the solution was evaporated, and the rest was dried in a vacuum for 24 h to provide the desired product as a yellow solid (0.768 g; 77%). m.p. 84–86 °C; [α]_D_^25^ = 48.3 ± 5.0 (c = 2 mgmL^−1^ in methanol); ^1^H-NMR (400.13 MHz, CD_3_OD) δ = 7.64 (s, 1H), 7.56 (s, 1H), 7.27 (d, 2H, *J* = 8.2 Hz), 6.74 (d, 2H, *J* = 8.4 Hz), 5.00 (d, 1H, *J*_1_ = 5.9 Hz), 4.74 (s, 1H), 4.30 (d, 1H, *J*_1_ = 5.9 Hz), 4.25 (1, 2H, *J* = 7.3 Hz), 3.77 (bs, 1H), 3.92 (s, 3H), 3.73, (bs, 1H), 3.43 (bs, 1H), 3.35 (s, 1H, s), 1.55–1.48 (m, 6H); 1.22 (s, 3H) ppm; ^13^C-NMR (100.62 MHz, CD_3_OD) δ = 175.57, 175.15, 174.84, 141.24, 129.83, 129.12, 128.50, 124.96, 123.31, 77.12, 66.67, 60.18, 60.11, 59.54, 46.03, 36.46, 27.78, 27.47, 15.63 ppm; IR (KBr): υ = 3461, 2921, 2852, 1706, 1688,1656, 1636, 1560, 1541, 1508, 1461, 1403, 1348, 1260, 1170, 1130, 673, 620, 474, 422 cm^−1^; (ESI^+^) *m/z* calculated for C_6_H_11_N_2_^+^: 111.1, found 111.0; (ESI^−^) *m/z* calculated for C_16_H_21_N_4_O_5_S^−^ 381.1, found 380.8.

##### Preparation of [P_6,6,6,14_][*seco*-Amx]

Trihexyl(tetradecyl)phosphonium chloride (1.042 g; 2.01 mmol) was dissolved in methanol and passed through an Amberlite IRA-400-OH ion-exchange column [18,99] (5 eq., flux rate 0.133 mLmL^−1^min^−1^ = 8 BVh^−1^). Then, the trihexyl(tetradecyl)phosphonium hydroxide solution that was formed was slowly added to amoxicillin (0.988 g; 2.36 mmol) that was dissolved in a 1.0 M aqueous ammonium solution (50 mgmL^−1^). The mixture was stirred at room temperature for 1 h. After solvent evaporation, the residue was dissolved in a 20 mL solution (methanol/acetonitrile 1:9) [18,99] and left refrigerated overnight (4 °C) [18,99] to induce the precipitation of the excess reagent. Then, the reagent crystals were filtered out, the solution was evaporated, and the rest was dried in a vacuum for 24 h to provide the desired product as a yellow viscous liquid (1.586 g; 92%). [α]_D_^25^ = 22.0 ± 5.8 (c = 2 mgmL^−1^ in methanol); (400.13 MHz, CD_3_OD) δ = 7.28 (d, 2H, *J* = 8.4 Hz), 6.76 (d, 2H, *J* = 8.4 Hz), 5.00 (d, 1H, *J* = 5.9 Hz), 4.53 (s, 1H), 4.31 (d, 1H, *J* = 5.9 Hz), 3.42 (s, 1H), 2.23–2.16 (m, 8H), 1.60–1.22 (m, 54H), 0.95–0.88 (m, 12H) ppm; ^13^C-NMR (100.62 MHz, CD_3_OD) δ = 175.35, 174.22, 142.01, 129.62, 128.92, 116.59, 77.12, 66.57, 60.17, 54.94, 43.76, 33.18, 32.27, 31.93, 31.56, 30.89, 30.58, 30.01, 27.96, 23.85, 23.57, 22.45, 19.62, 19.15, 14.58, 14.46 ppm; IR (KBr): ν = 3419, 3921, 2107.38, 1638, 1560, 1506, 1459, 1398, 1270, 1130, 1000, 668, 619, 570, 476, 456, 433, 412 cm^−1^; (ESI^+^) *m/z* calculated for C_32_H_68_P^+^: 483.8 found 483.6; (ESI^−^) *m/z* calculated for C_16_H_21_N_4_O_5_S^−^ 381.1, found 381.0.

##### Preparation of [C_16_Pyr][*seco*-Amx]

Cetylpyridinium chloride (0.456 g; 1.28 mmol) was dissolved in methanol and passed through an Amberlite IRA-400-OH ion-exchange column [18,99] (5 eq., flux rate 0.133 mLmL^−1^min^−1^ = 8 BVh^−1^). Then, the cetylpyridinium hydroxide solution that was formed was slowly added to amoxicillin (0.587 g; 1.40 mmol) that was dissolved in a 1.0 M aqueous ammonium solution (50 mgmL^−1^). The mixture was stirred at room temperature for 1 h. After solvent evaporation, the residue was dissolved in a 20 mL solution (methanol/acetonitrile 1:9) [18,99] and left refrigerated overnight (4 ºC) [18,99] to induce the precipitation of the excess reagent. When the reagent crystals were filtered out, the solution was evaporated, and the rest was dried in a vacuum for 24 h to provide the desired product as a yellow solid (0.402 g; 52%). m.p. 96–98 °C; [α]_D_^25^ = 77.0 ± 5.8 (c = 2 mgmL^−1^ in methanol); ^1^H-NMR (400.13 MHz, CD_3_OD) δ = 8.98 (d, 2H, *J* = 5.8 Hz), 8.58 (t, 1H, *J* = 7.74 Hz), 8.10 (t, 2H, *J* = 6.7 Hz), 7.26 (d, 2H, *J* = 8.5 Hz), 6.73 (d, 2H, *J* = 8.4 Hz), 5.01 (d, 1H, *J* = 6.0 Hz), 4.62 (t, 2H, *J* = 7.5 Hz), 4.46 (s, 1H), 4.30 (d, 1H, *J* = 6.0 Hz), 3.44 (s, 1H), 2.02, (t, 2H, *J* = 6.9 Hz), 1.48 (s, 3H), 1.38–1.26 (m, 28H), 1.22 (s, 3H) 0.90 (3H, t, *J* = 6.7 Hz) ppm; ^13^C-NMR (100.62 MHz, CD_3_OD) δ = 176.32, 175.60, 174.94, 158.32, 150.28, 146.87, 145.91, 132.86, 129.54, 116.55, 77.13, 66.61, 63,17, 60.14, 33.11, 32.53, 30.80, 30.67, 30.52, 30.16, 27.24, 23.77, 14.49 ppm; IR (KBr): υ = 3440, 2914, 2849, 1685, 1651, 1636, 1560, 1488, 1472, 1400, 1384, 1260, 1175, 1128, 847, 778, 720, 687, 621, 498, 476 cm^−1^; (ESI^+^) *m/z* calculated for C_21_H_38_N^+^: 304.3 found 304.4; (ESI^−^) *m/z* calculated for C_16_H_21_N_4_O_5_S^−^ 381.1, found 380.9.

##### Preparation [choline][*seco*-Amx]

(2-hydroxyethyl)trimethylammonium chloride (0.179 g; 1.28 mmol) was dissolved in methanol and passed through an Amberlite IRA-400-OH ion-exchange column [18,99] (5 eq., flux rate 0.133 mLmL^−1^min^−1^ = 8 BVh^−1^). Then, the hydroxide solution that was formed was slowly added to amoxicillin (0.587 g; 1.40 mmol) that was dissolved in a 1.0 M aqueous ammonium solution (50 mgmL^−1^). The mixture was stirred at room temperature for 1 h. After solvent evaporation, the residue was dissolved in a 20 mL solution (methanol/acetonitrile 1:9) [18,99] and left refrigerated overnight (4 °C) [18,99] to induce the precipitation of the excess reagent. When the reagent crystals were filtered out, the solution was evaporated, and the rest was dried in a vacuum for 24 h to provide the desired product as a yellow solid (0.570 g; 93%). m.p. 143–144 °C; [α]_D_^25^ = 104.0 ± 3.4 (c = 2 mgmL^−1^ in methanol); ^1^H-NMR (400.13 MHz, CD_3_OD) δ = 7.36 (d, *J* = 8.1 Hz, 2H), 6.93 (d, *J* = 8.1 Hz, 2H), 5.06 (d, *J* = 6.8 Hz, 1H), 4.64 (s, 1H), 4.40 (d, *J* = 6.8 Hz, 1H), 4.13–4.04 (m, 2H), 3.53 (t, *J* = 4.6 Hz, 2H), 3.37 (s, 1H), 3.22 (s, 9H), 1.46 (s, 3H), 1.24 (s, 3H) ppm; ^13^C-NMR (100.62 MHz, CD_3_OD) δ = 176.14, 175.48, 174.46, 156.70, 131.47, 129.19, 116.54, 75.51, 67.98, 65.21, 59.78, 58.63, 58.46, 56.16, 54.48, 54.44, 54.40, 26.98, 26.79 ppm; IR (KBr): υ = 3300, 2964, 2927, 1673, 1594, 1513, 1435, 1389, 1251, 1118, 1130, 1087, 956, 837, 780 cm^−1^.

##### Preparation of [C_2_OHMIM][*seco*-Amx]

3-(2-hydroxyethyl)-1-methylimidazolium chloride (0.456 g; 1.28 mmol) was dissolved in methanol and passed through an Amberlite IRA-400-OH ion-exchange column [18,99] (5 eq., flux rate 0.133 mLmL^−1^min^−1^ = 8 BVh^−1^). Then, the hydroxide solution that was formed was slowly added to amoxicillin (0.525 g; 1.44 mmol) that was dissolved in a 1.0 M aqueous ammonium solution (50 mgmL^−1^). The mixture was stirred at room temperature for 1 h. After solvent evaporation, the residue was dissolved in a 20 mL solution (methanol/acetonitrile 1:9) [18,99] and left refrigerated overnight (4 °C) [18,99] to induce the precipitation of the excess reagent. When the reagent crystals were filtered from the solution, the solution was evaporated, and the rest was dried in a vacuum for 24 h to provide the desired product as a yellow solid (0.359 g; 60%). m.p. 109–111 °C; [α]_D_^25^ = 47.3 ± 3.6 (c = 2 mgmL^−1^ in methanol); ^1^H-NMR (400.13 MHz, CD_3_OD) δ = 7.61 (s, 1H), 7.55 (s, 1H), 7.27 (d, 2H, *J* = 8.4 Hz), 6.74 (d, 2H, *J* = 8.4 Hz), 5.00 (d,1H, *J* = 6.0 Hz), 4.47 (s, 1H), 4.30–4.27 (m, 3H), 3.92 (s, 3H), 3.86 (t, 2H, *J* = 4.86 Hz), 3.43 (s, 1H), 1.48 (s, 3H), 1.22 (s, 3H) ppm; ^13^C-NMR (100.62 MHz, CD_3_OD) δ = 176.33, 175.62, 174.95, 158.34, 132.89, 129.54. 124.75,124.04, 116.56, 77.17 66.64, 61.11, 60.15, 59.83, 59.50, 53.81, 36.45, 27.78, 27.47 ppm; IR (KBr): υ = 3420, 2970, 2921, 1722, 1690, 1655, 1599, 1577, 1545, 1509, 1436, 1386, 1322, 1251, 1170, 1132, 1108, 1067, 877, 840, 820, 778, 652, 621, 535, 474 cm^−1^; (ESI^+^) *m/z* calculated for C_6_H_11_N_2_O^+^: 127.2, found 127.0; (ESI^−^) *m/z* calculated for C_16_H_21_N_4_O_5_S^−^ 381.1, found 380.9.

## Figures and Tables

**Table 1:** Yield, physical state, and melting point of the synthesized API-OSILs.

Compound	Yield	Physical State	Melting Point/°C
[EMIM][*seco*-Amx]	77%	Yellow solid	84–86
[C_2_OHMIM][*seco*-Amx]	60%	Yellow solid	109–111
[P_6,6,6,14_][*seco*-Amx]	92%	Yellow viscous liquid	-
[C_16_Pyr][*seco*-Amx]	94%	Yellow solid	96–98
[Choline][*seco*-Amx]	93%	Yellow solid	143–144
[Na][*seco*-Amx]	96%	Yellow solid	137–139
[EMIM][*seco*-Pen]	81%	Colorless viscous liquid	
[C_2_OHMIM][*seco*-Pen]	83%	Yellow solid	48–50
[Choline][*seco*-Pen]	95%	Yellow solid	69–71
[P_6,6,6,14_][*seco*-Pen]	97%	Yellow viscous liquid	-
[C_16_Pyr][*seco*-Pen]	89%	Yellow solid	76–78
[TEA][*seco*-Pen]	90%	Yellow viscous liquid	-
[K][*seco*-Pen]	97%	White solid	193–195

* This is a outside table footnote.

**Table 2:** Minimum inhibitory concentrations (mM) and relative decrease of inhibitory concentrations (RDIC) of the new compounds that were produced on sensitive strains.

Compound	*S. aureus* ATCC25923	*RDIC*	*E. coli* ATCC25922	*RDIC*
[EMIM][*seco*-Amx]	5.0	0.01	2.5	0.002
[C_2_OHMIM][*seco*-Amx]	0.050	1	5.0	0.001
[P_6,6,6,14_][*seco*-Amx]	>5.0	<0.01	0.5	0.01
[C_16_Pyr][*seco*-Amx]	>5.0	<0.01	0.050	0.1
[Choline][*seco*-Amx]	>5.0	<0.01	>5.0	<0.001
Na[*seco*-Amx]	>5.0	<0.01	>5.0	<0.001
Amx	0.050	1	0.005	1
[EMIM][*seco*-Pen]	>5.0	<0.1	>5.0	<0.1
[C_2_OHMIM][*seco*-Pen]	**0.005**	**100**	>5.0	<0.1
[Choline][*seco*-Pen]	>5.0	<0.1	5.0	0.1
[P_6,6,6,14_][*seco*-Pen]	>5.0	<0.1	>5.0	<0.1
[C_16_Pyr][*seco*-Pen]	>5.0	<0.1	>5.0	0.1
[TEA][*seco*-Pen]	>5.0	<0.1	**0.050**	**10**
K[*seco*-Pen]	>5.0	<0.1	>5.0	<0.1
K[Pen]	0.500	1	0.500	1
[EMIM][Br]	0.05	---	>5	---
[C_2_OHMIM][Cl]	>5.0	---	5.0	---
[P_6,6,6,14_][Cl]	2.5	---	2.5	---
[C_16_Pyr][Cl]	0.5	---	0.5	---
[Choline][Cl]	2.5	---	>5.0	---
[TEA][Br]	2.5	---	>5.0	---

**Table 3:** Minimum inhibitory concentrations (mM) and and relative decrease of inhibitory concentration (RDIC) of the new compounds that were produced on resistant strains.

Compound	*E. coli* CTX M9	*RDIC*	*E. coli* CTX M2	*RDIC*	MRSA ATCC 43300	*RDIC*
[EMIM][*seco*-Amx]	>5	-	>5	-	>5	-
[C_2_OHMIM][*seco*-Amx]	>5	-	> 5	-	5	>1
[P_6,6,6,14_][*seco*-Amx]	0.05	>100	1.0	>5	> 5	-
[C_16_Pyr][*seco*-Amx]	0.05	>100	0.05	>100	0.005	>1000
[Choline][*seco*-Amx]	0.5	>10	0.05	>100	0.5	10
Na[*seco*-Amx]	>5	-	>5	-	>5	-
Amx	>5	1	>5	1	>5	1
[EMIM][*seco*-Pen]	>5	-	>5	-	>5	-
[C_2_OHMIM][seo-Pen]	>5	-	>5	-	>5	-
[Choline][*seco*-Pen]	1.0	>5	>5	-	1.0	>5
[P_6,6,6,14_][*seco*-Pen]	0.5	>10	0.5	>10	>5	-
[C_16_Pyr]*seco*-[Pen]	0.5	>10	0.5	>10	0.05	>100
[TEA][*seco*-Pen]	>5	-	> 5	-	>5	-
K[*seco*-Pen]	>5	-	>5	-	>5	-
K[Pen]	>5	1	>5	1	>5	1
